# Communicative Development Inventories (CDIs) in etiologically diverse developmental conditions: A systematic review

**DOI:** 10.1016/j.ridd.2026.105256

**Published:** 2026-02-17

**Authors:** Zuzanna Laudańska, Patrice van der Venne, Helena Preis, Steffi Sachse, Christian P. Schaaf, Jeremy I. Borjon, Hana D’Souza, Daniel Holzinger, Ewa Haman, Nivedita Mani, Luise Poustka, Dajie Zhang, Peter B. Marschik

**Affiliations:** a Department of Child and Adolescent Psychiatry and Psychotherapy, Heidelberg University Hospital, Heidelberg University, Heidelberg, Germany; b Institute of Psychology, Polish Academy of Sciences, Warsaw, Poland; c Institute of Psychology, Heidelberg University of Education, Heidelberg, Germany; d Institute of Human Genetics, Heidelberg University Hospital, Heidelberg University, Heidelberg, Germany; e Department of Psychology, University of Houston, USA; f Texas Institute for Measurement, Evaluation, and Statistics, University of Houston, USA; g Texas Center for Learning Disorders, University of Houston, USA; h Centre for Human Developmental Science, School of Psychology, Cardiff University, UK; i Clinical Research Institute for Developmental Medicine, Johannes Kepler University Linz, Austria; j Faculty of Psychology, University of Warsaw, Warsaw, Poland; k Georg-Elias-Müller Institute for Psychology, Department for Psychology of Language, University of Göttingen, Göttingen, Germany; l iDN – interdisciplinary Developmental Neuroscience, Department of Neurology, Medical University of Graz, Austria; m Center of Neurodevelopmental Disorders (KIND), Department of Women’s and Children’s Health, Centre for Psychiatry Research, Karolinska Institutet & Region Stockholm, Stockholm, Sweden; n Child and Adolescent Psychiatry and Psychotherapy, University Medical Center Göttingen, German Center for Child and Adolescent Health (DZKJ), Göttingen, Germany

**Keywords:** Communicative Development Inventories, CDIs, Language, Autism, Down syndrome, Williams syndrome, Cerebral palsy, Angelman syndrome, DDX3X syndrome, 5p deletion syndrome, Fragile X syndrome, Genetic conditions

## Abstract

The MacArthur-Bates Communicative Development Inventories (CDIs) are widely used parent-report tools for assessing early language development, including gesture use, expressive and receptive vocabulary, and early morpho-syntactic capacities. While originally developed for typically developing children aged 8 up to 36 months and aimed at detecting developmental language disorder, CDIs have been increasingly applied in studies of neurodevelopmental and genetic conditions, where language development often diverges from typical trajectories. In this review, we synthesize literature on the use of CDIs in a range of clinical populations, including autism, Down syndrome, Williams syndrome, cerebral palsy, Angelman syndrome, DDX3X syndrome, 5p deletion syndrome, fragile X syndrome, and others. We highlight condition-specific patterns of expressive vocabulary development, discuss the value of longitudinal data collection using CDIs, and visualize age trends that capture change and variability across developmental pathways. Particular attention is given to methodological considerations such as cross-linguistic adaptations, reporting biases, and the limitations of single-timepoint assessments. While CDIs show promise for tracking language trajectories and informing early support, challenges remain in ensuring their reliability, validity, and suitability as screening tools. We conclude by emphasizing the importance of longitudinal, cross-condition, and cross-cultural approaches to better understand atypical language development and to improve the utility of CDIs in both research and applied settings.

## 1. Introduction

Language development during infancy and toddlerhood can be studied using a number of methods spanning from parent based reporting to experimenter-led standardized testing and home-based daylong audio recordings with automatic calculation of outcomes (e.g., Laudańska et al., 2025). Parental reports are particularly useful for identifying early language delays, as reduced vocabulary size, the absence of multi-word utterances, and slow syntax acquisition are common ‘red flags’ that often prompt parents to seek specialist evaluation (Guinchat et al., 2012; Herlihy et al., 2015; Richards et al., 2016; Riva et al., 2021). Prominent and widely used parent-report tools are the MacArthur-Bates Communicative Development Inventories (CDIs, Fenson et al., 1993; Marchman et al., 2023) that assess early language acquisition, including vocabulary comprehension, production, gesture use, and grammar (Fenson et al., 2006). As of 2025, CDIs have been adapted into more than 100 languages (https://mb-cdi.stanford.edu/, e.g., Bleses et al., 2008; Bornstein et al., 2004; Eriksson et al., 2012; Jarůšková et al., 2023) which has allowed for cross-linguistic studies of language development. Most language versions have two age-dependent long forms ‘Words and Gestures’ (CDI-WG; 8–18 months) and ‘Words and Sentences’ (CDI-WS; 16- up to 36 months). The form for younger children (CDI-WG) usually contains around 400 words (e.g., 396 for American English, but 428 for Hebrew), while the form for older children contains around 680 (in American English version), up to 710 words (Mandarin version) which are organized into semantic categories. There are also short versions available for some languages that include circa 100 words (Fenson et al., 2000) and are used for rapid language assessment (screening) when detailed information is not required as well as CDI-III forms for assessing language development beyond 36 months of age (Eriksson, 2017; Marchman & Dale, 2023). Each version consists of an extensive checklist where the specific lexical items that the infant understands and/or produces can be ticked off (Fenson et al., 2007). In the CDIs, the total number of words understood (receptive vocabulary; score used in versions for younger children from age 8–18 months) and spoken (expressive vocabulary; score used in all age versions) are calculated as raw scores that can also be transformed into percentiles for further comparisons (if norms are available for a given language). In the case of repeated measurements in longitudinal designs, the developmental growth curves of the number of words spoken by the child can be assessed and compared with the growth curves for different languages available in Wordbank (https://wordbank.stanford.edu, Frank et al., 2021).

The CDIs have been validated and studied across diverse populations, including typically developing children (TD) and those with neurodevelopmental or genetic disorders. In typical development, they are widely used for tracking language acquisition from 8 up to 36 months of age (the upper age limit depending on the language version, Dale & Fenson, 1996; Marchman & Dale, 2023; Rescorla et al., 2011). In children with neurodevelopmental conditions, CDIs are often used beyond the standard age range because language development may be delayed or follow an atypical trajectory (Berglund et al., 2001; Charman et al., 2003; Deckers et al., 2016). Consequently, CDI scores are typically interpreted relative to the child’s developmental language level rather than chronological age (Bello et al., 2014; Luyster et al., 2007; Zampini and D’Odorico, 2013). This approach enables comparison with typically developing peers at a similar developmental stage, but it complicates direct age-based comparisons.

For many years now, the CDIs have been thought to have the potential to identify children with delays in early language development (e.g., Feldman et al., 2005; Rescorla et al., 2011), as a tool for monitoring changes in speech-language skills or predicting future language capacities (Can et al., 2013), and as outcome measure (e.g., D’Souza et al., 2015). Deviations in speech-language skills are co-occurring with many neurodevelopmental and genetic disorders such as autism, Down syndrome, and cerebral palsy. These conditions are characterized by speech-language and communicative challenges, such as delays or deviations in receptive and expressive vocabulary development, atypical gesture use, and difficulties with social communication (Abbeduto et al., 2007; Tager-Flusberg et al., 2009a,b). Over the past decades, CDIs have been instrumental in characterizing the language phenotypes associated with various disorders (Luyster et al., 2008; Rescorla et al., 2011) and proven valuable in both research and applied settings. At the same time, several methodological concerns have been raised regarding their use in atypical populations, including variability in test–retest stability and predictive validity (Feldman et al., 2000), differential reporting biases across clinical groups (Galeote et al., 2016; Marschik, 2014; Miller et al., 1995), and limited comparability across languages, CDI versions, and developmental profiles (Eriksson, 2017; Jarůšková et al., 2023). These issues are particularly relevant for studying neurodevelopmental and genetic conditions, where disorder specific developmental profiles often require extending CDI use beyond standard age norms, complicating cross-condition and cross-linguistic comparisons. This review aims to synthesize and contextualize existing applications of the CDIs in assessing expressive vocabulary development in neurodevelopmental conditions and genetic syndromes. By integrating findings from the literature, we provide a descriptive overview of early language trajectories across clinical groups, highlight divergent patterns of language acquisition and identify methodological challenges that limit cross-condition and cross-linguistic comparability. We further underscore the importance of early, reliable language assessment tools in advancing our understanding of atypical language trajectories which may lead to provision of tailored support for affected children. Our focus lies on expressive vocabulary because production events are directly observable to caregivers and are aligned with the most frequent clinical red flags that prompt referral to specialists. In presenting condition-by-condition profiles we also shed light on alternative and augmentative communication strategies strengthening classical parental checklist assessments.

## 2. Methods

To provide a comprehensive, systematic review of the empirical literature on the use of the CDIs regarding expressive vocabulary development in neurodevelopmental conditions and genetic syndromes, we conducted a database search following PRISMA guidelines (Page et al., 2021). We aimed to examine the developing mental lexicon within conditions that show broad, multi-domain atypical development. Accordingly, we did not include language-specific conditions (e.g., developmental language disorder, familial risk of developmental dyslexia), whose primary impairments are confined to the language domain.

### 2.1. Search strategy

To identify studies of interest, we searched PubMed and Web of Science, applying database-specific terms:

- For PubMed we used the baseline inquiry “((((((“Communicative Development Inventories”) OR (“Communicative Development Inventory”)) OR (MB-CDI)) OR (ELFRA)) OR (FRAKIS))”, adding disorder-specific search terms: cerebral palsy (*“AND (ALL= (Cerebral Palsy))”*), autism (*“AND (ALL*=*(Autism)* || *ALL*= *(ASD)))”*), genetic disorders (separate searches conducted for: *“AND (ALL*=(genetic*))”, *“AND (ALL*=*(syndrome))”*, *“AND (ALL*=*(Down syndrome)* || *ALL*= *(trisomy)))”*), and ADHD (*“AND (ALL*= *(attention deficit hyperactivity disorder)* || *ALL*= *(ADHD)))”*).
- For Web of Science we used the baseline inquiry “*((((((ALL*=*(“Communicative Development Inventories”)) OR ALL*= *(“Communicative Development Inventory”)) OR ALL*= *(MB-CDI)) OR ALL*= *(ELFRA)) OR ALL*= *(FRAKIS))*”, subsequently adding disorder-specific search terms, such as: cerebral palsy (*“AND (ALL*=*(Cerebral Palsy) OR ALL*= *(CP)))”*), autism (*“AND (ALL*=*(Autism) OR ALL*= *(ASD)))”*), genetic disorders (separate searches conducted for: *“AND (ALL*=(genetic*))”, *“AND (ALL*=*(syndrome))”*, *“AND (ALL*=*(Down syndrome) OR ALL*= *(trisomy)))”*), and ADHD (*“AND (ALL*=*(attention deficit hyperactivity disorder) OR ALL*= *(ADHD)))”*).

### 2.2. Selection criteria

#### 2.2.1. Title-abstract review

The exclusion criteria for the first selection phase were: (a) systematic reviews, meta-analysis or dissertations; (b) no reference to and/or analysis of the above-mentioned conditions; (c) no reference to and/or analysis using the CDIs; (d) paper not in English.

#### 2.2.2. Full-text review

Full texts of all studies that passed the title-abstract review phase were retrieved and split between two authors (HP, PVDV), who applied the abovementioned exclusion criteria.

#### 2.2.3. Study selection

**Fig. 1.**
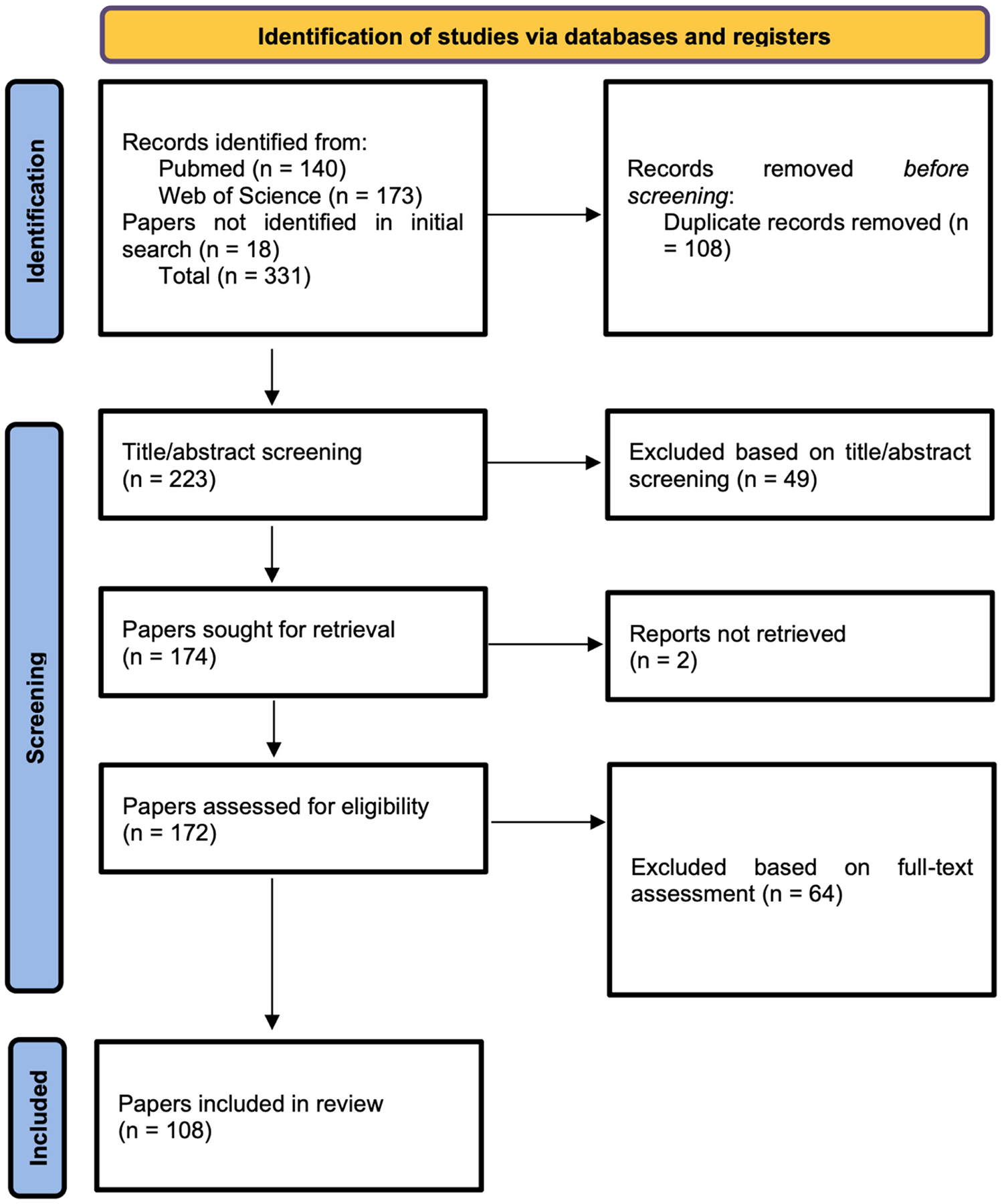
PRISMA Flow diagram.

The systematic search strategy yielded a total of 313 publications in the initial search phase (see Fig. 1). After removing duplicates, 205 publications were screened in at the first stage. The title-abstract review eliminated 49 publications, leaving 156 for a full-text review. Out of those, two papers could not be retrieved, leaving 154 for data extraction and in-depth review. During the full-text review, another 64 papers were excluded, applying the above-mentioned criteria, resulting in 90 publications included in the final systematic review.

**Fig. 2.**
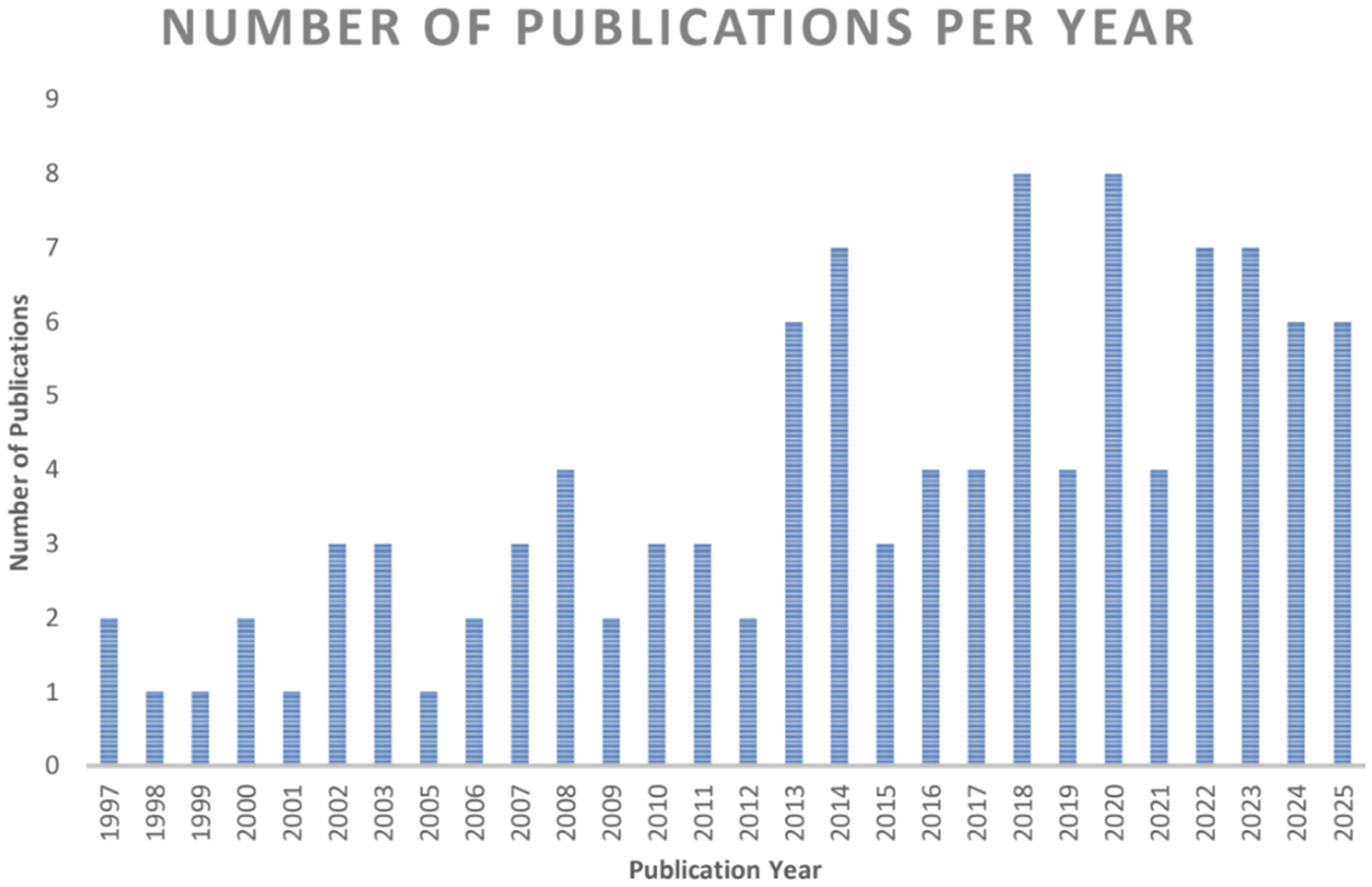
Number of publications per year mentioning the use of CDI in studies on children with NDDs or genetic disorders.

**Fig. 3.**
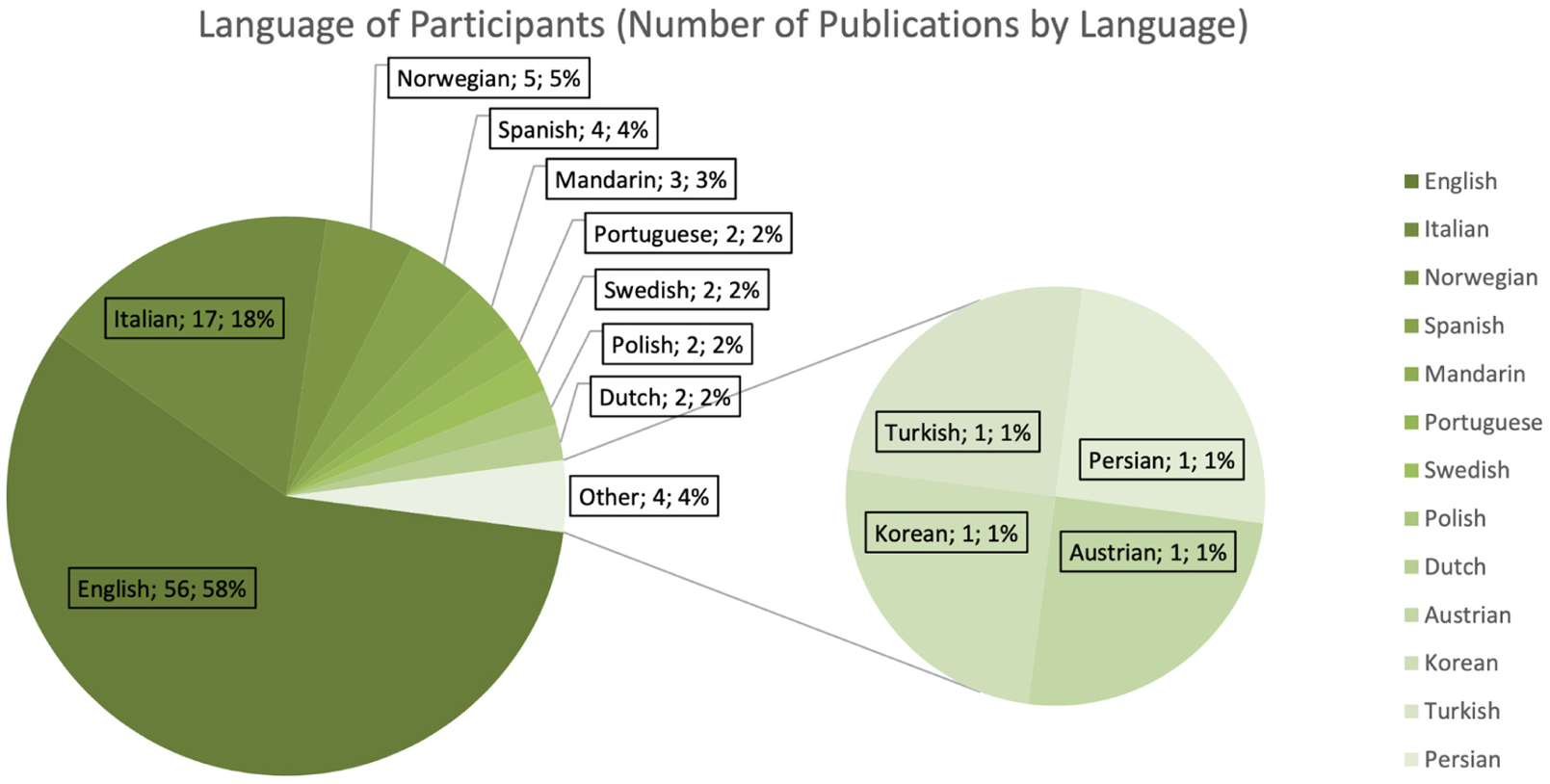
Number of publications based on the language spoken by participants and the CDI-Version used (Total number & percentages).

**Fig. 4.**
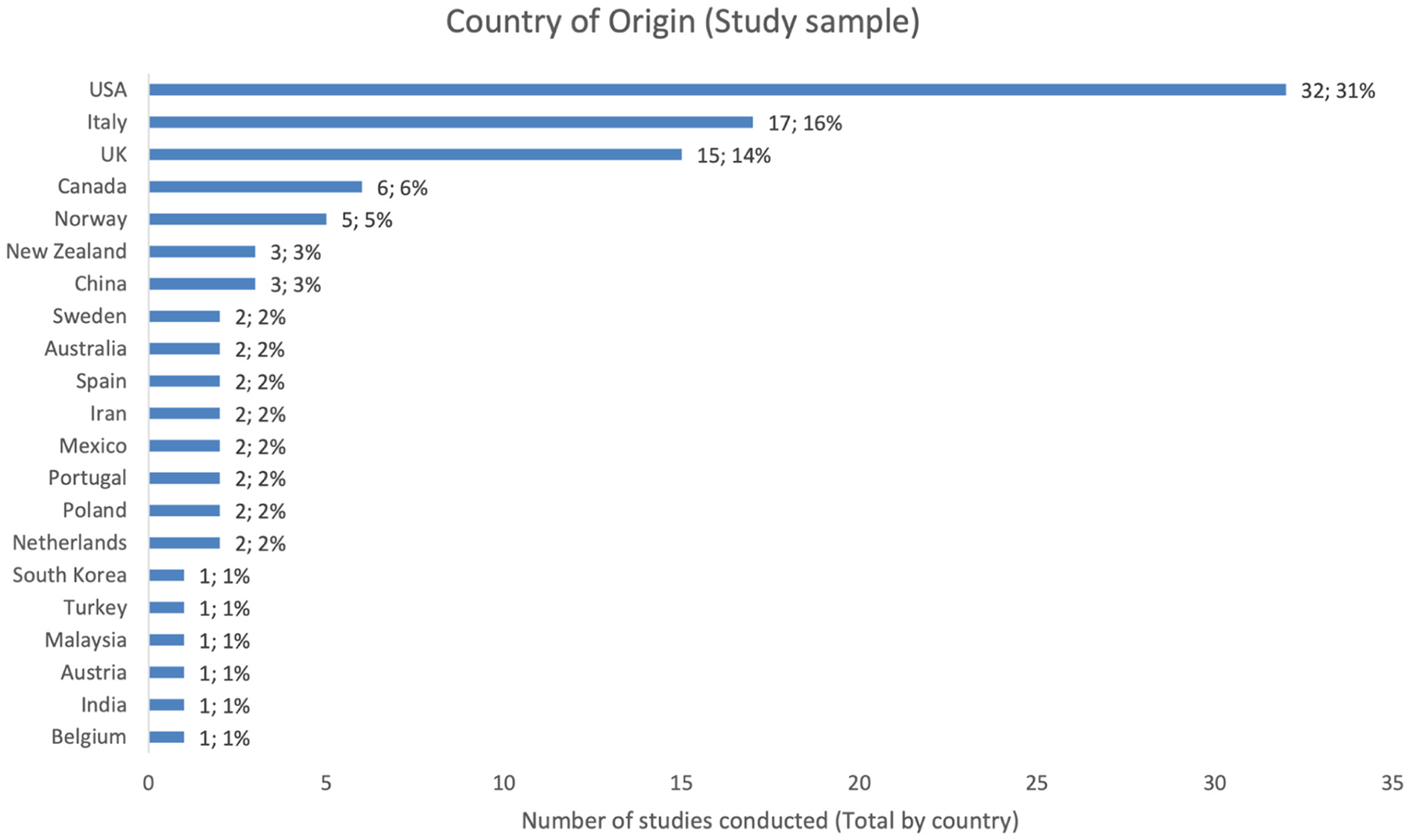
Number of publications by country of study origin (Total number & percentages).

**Fig. 5.**
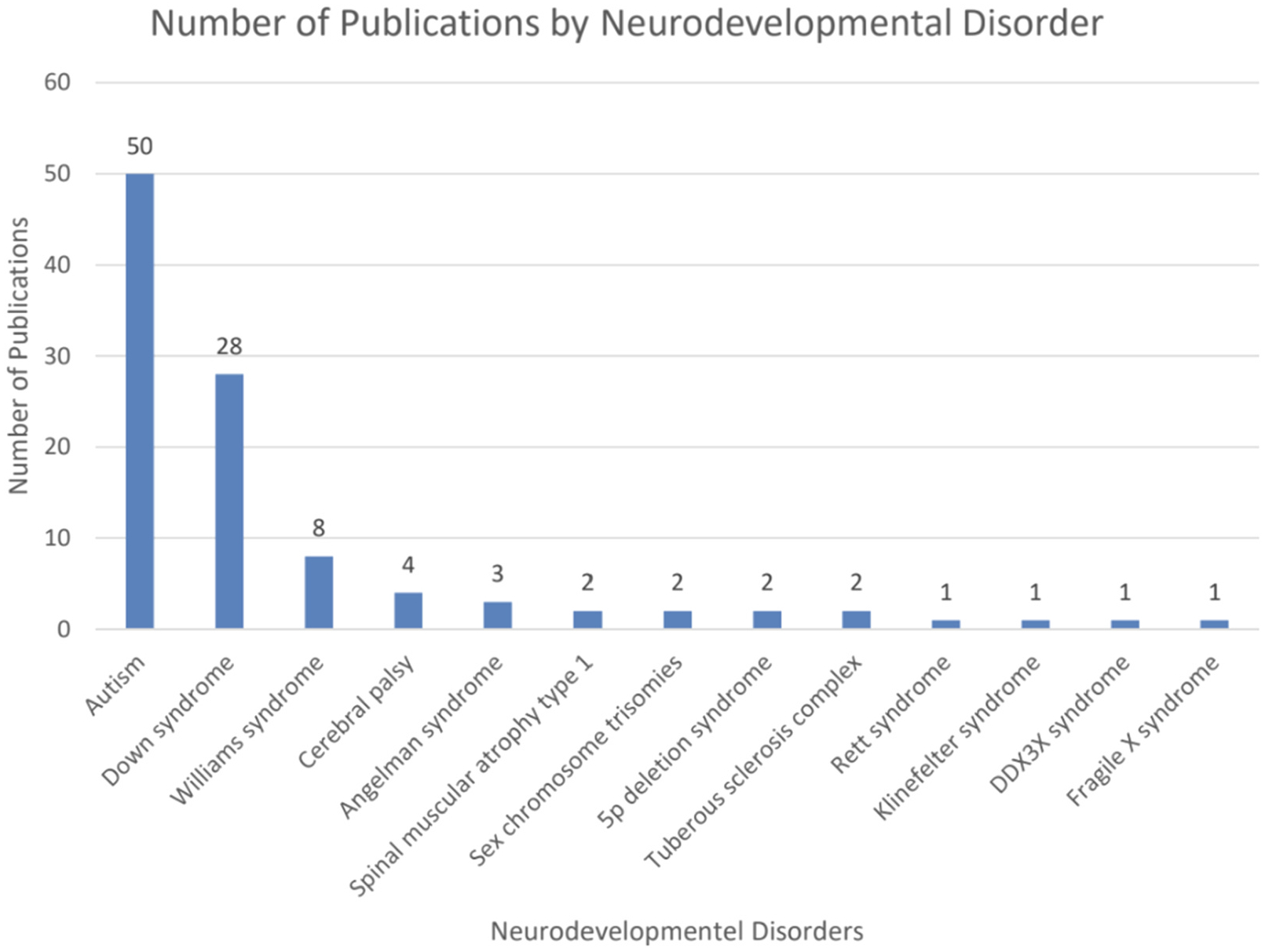
Number of publications by condition.

Additional 18 publications were added through snowballing, ancestral research and follow-up searches by another co-author (ZL). These publications were likely not captured by the initial search due to different names for the CDIs used (for example, using names or abbreviations in languages other than English such as “Il Primo Vocabolario del Bambino”, Caselli & Casadio, 1995, PVB in Italian), or because none of the search terms were indicated as paper keywords. These papers were added subsequently and assessed using the same criteria as mentioned prior. Based on this systematic search, we have analyzed the number of publications per year mentioning the use of CDI in studies on children with NDDs or genetic disorders (Fig. 2), the language spoken by participants (Fig. 3), the country of study origin (Fig. 4) and the number of publications by condition (Fig. 5).

#### 2.2.4. Calculation of condition-specific age trends

**Fig. 6.**
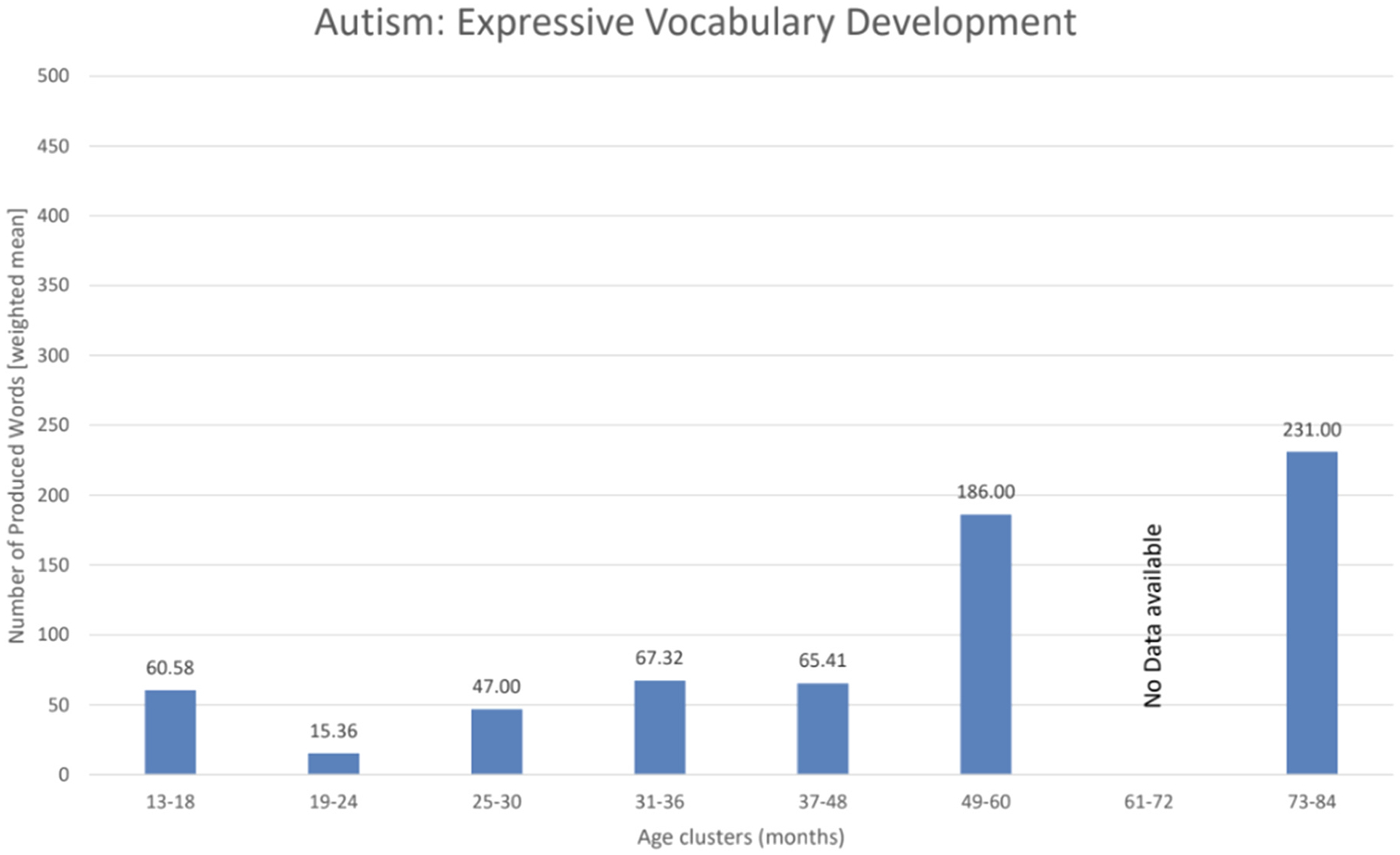
Autism-specific age trends (including samples with autism diagnoses or elevated likelihood of autism) were visualized using weighted mean expressive vocabulary scores grouped into age clusters. Six-month intervals were used between 13 and 36 months to capture rapid early developmental change (e.g., 13–18 months), whereas 12-month intervals were applied beyond 36 months due to sparser data (e.g., 73–84 months). Studies were assigned to clusters based on the mean age of their sample, and weighted mean vocabulary scores within each cluster determined the bar heights.

**Fig. 7.**
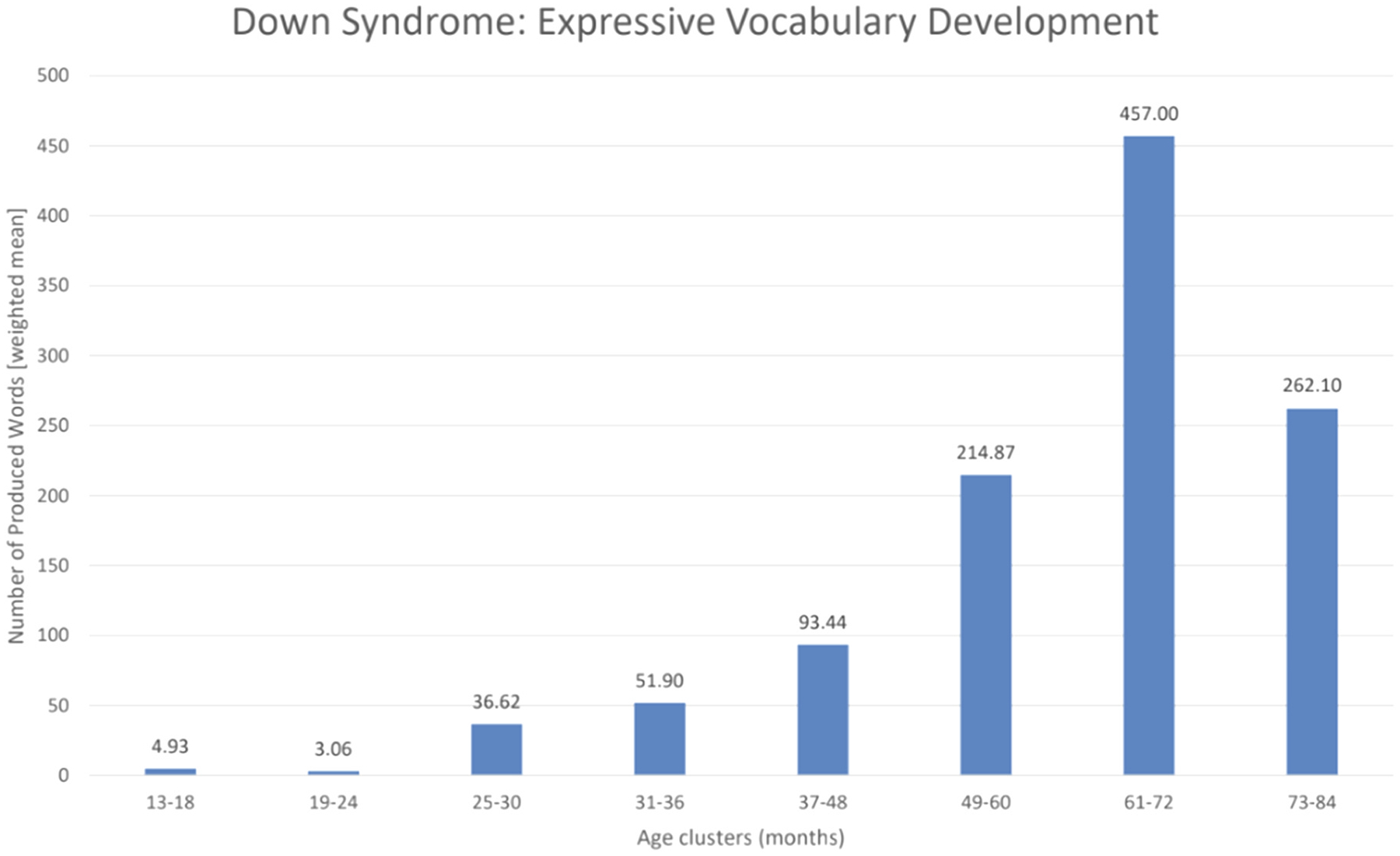
Down syndrome–specific age trends in expressive vocabulary based on weighted mean CDI scores grouped into age clusters. Six-month intervals were used between 13 and 36 months to capture rapid early developmental change (e.g., 13–18 months), and 12-month intervals thereafter due to sparser data beyond early toddlerhood (e.g., 73–84 months). Studies were assigned to clusters based on the mean age of the sample, and weighted mean vocabulary scores within each cluster determine bar height.

**Fig. 8.**
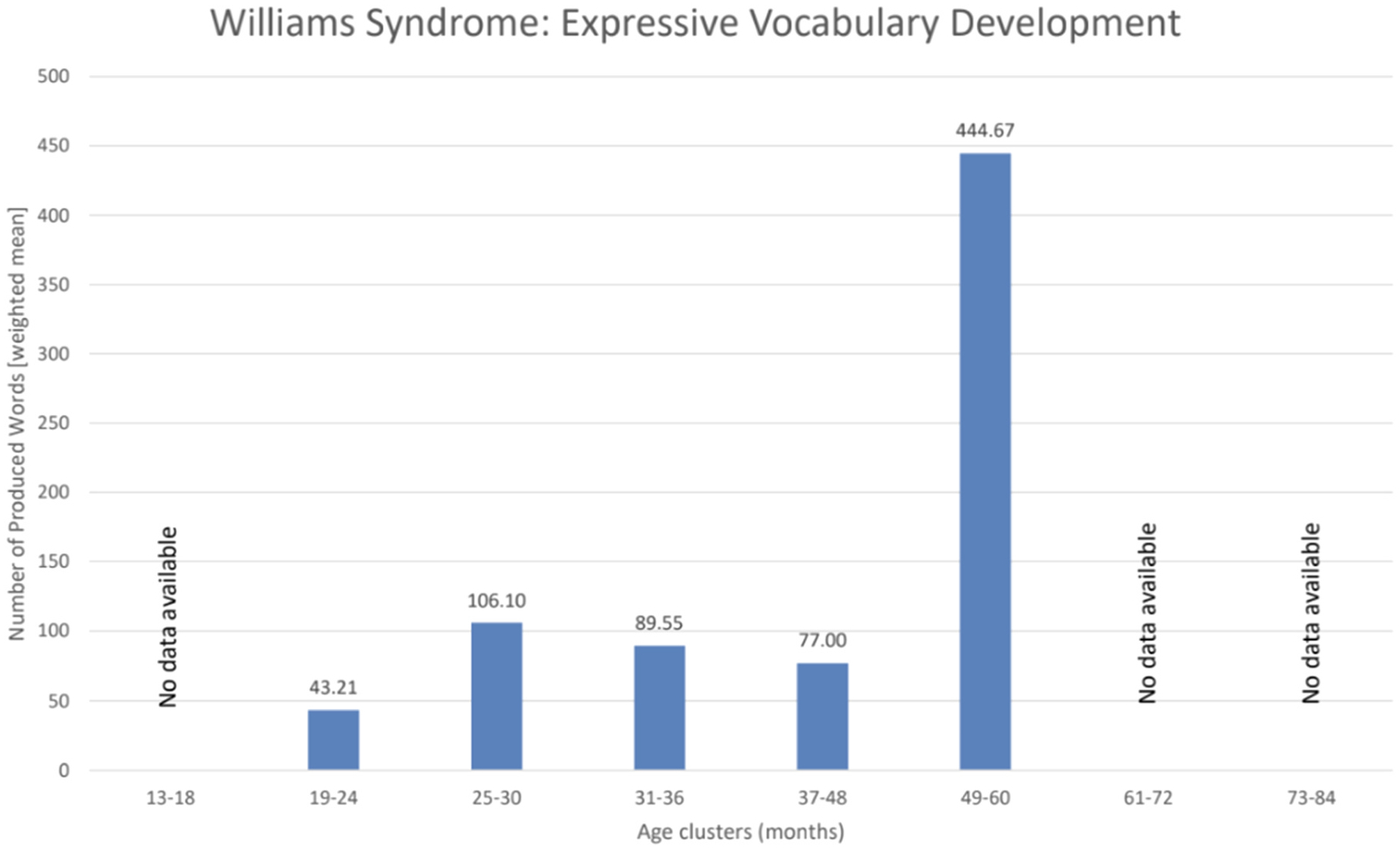
Williams syndrome–specific age trends in expressive vocabulary based on weighted mean CDI scores grouped into age clusters. Six-month intervals were used between 13 and 36 months to capture rapid early developmental change (e.g., 13–18 months), and 12-month intervals thereafter due to sparser data beyond early toddlerhood (e.g., 73–84 months). Studies were assigned to clusters according to the mean age of the sample, and weighted mean vocabulary scores within each cluster determine bar height.

To display the condition-specific age trends (Figs. 6–8), we extracted data on the study sample sizes, the mean age of participants at the CDI assessments as well as the raw scores (mean and standard deviation) on expressive vocabulary from the papers. As such, only 25 studies assessing autism reported the necessary data to be included in the age trend visualizations. For autism, we chose to include data from study populations with an autism diagnosis, or at elevated likelihood due to an older sibling with an already existing autism diagnosis. Further, studies on minimally verbal or deaf participants were excluded, in order to avoid potentially misleading trends. Regarding Down syndrome, data from only 19 studies could be included. Finally, regarding Williams syndrome, data could be extracted from 8 studies. No other conditions yielded sufficient or usable data for inclusion in these analyses. We subsequently defined age ranges of six-month intervals for the first three years of life and 12-month intervals from the third year of life onwards. The six-month age ranges were chosen due to an uneven distribution of data as well as the importance of finer-grained intervals during the initial rapid development of infants. The shift to 12-month age bands after the third year was driven by the limited availability of data beyond early toddlerhood and an uneven distribution of longitudinal CDI data at older ages, which would otherwise have resulted in potentially misleading trends. To account for differences in sample sizes across the different condition-specific papers, we calculated weighted means using the following formula.


$$\text{Weighted}\;\text{Mean}=(({\text{X}}_{1}*{\text{Y}}_{1})+({\text{X}}_{2}*{\text{Y}}_{2})+\ldots +({\text{X}}_{\text{n}}*{\text{Y}}_{\text{n}}))/({\text{Y}}_{1}+{\text{Y}}_{2}+\ldots +{\text{Y}}_{\text{n}})$$

X refers to the mean raw scores for expressive vocabulary in the individual publications, and Y refers to the number of participants with a specific condition in the individual publications. Based on available data, condition-specific age trend visualizations were created for autism (Fig. 6), DS (Fig. 7), and WS (Fig. 8). All information taken from the publications was manually entered into Excel. All the figures and calculations they are based on were performed using Excel.

## 3. Results

### 3.1. Studies across countries and languages

The CDIs have been widely used in research on language development in neurodevelopmental conditions. From 1997 to August 2025 (last literature search), 108 publications reported using CDIs (Fig. 2), but 4 did not provide CDI-based results. Regarding participants’ geographic location and language, English-learning children in the U.S. and the U.K. were the most intensively studied, followed by children in Italy. Overall, studies included in our analysis described samples speaking 12 different languages in 21 countries (Figs. 3–4). Among the studies that reported CDI scores, the most studied neurodevelopmental condition was autism (48 % of studies; including infants at elevated likelihood who had an older sibling with autism), followed by Down syndrome (27 % of studies), Williams syndrome (8 % of studies) and cerebral palsy (4 % of studies) (Fig. 5).

### 3.2. Condition-specific age trend visualizations

Across all the conditions (please see Fig. 5) 37 papers did not report raw (mean) scores for expressive vocabulary. This resulted in data from 64 papers that could be used for presenting condition-specific age trend visualization (Figs. 6–8). Sixteen studies followed the same children in longitudinal designs, collecting CDI data at multiple timepoints or collecting it at one timepoint but with other speech-language measures at other ages (see Laudańska et al., in press, for more details on longitudinal findings).

## 4. Discussion

Here we reviewed the application of the Communicative Development Inventories (CDIs) in assessing expressive vocabulary development in neurodevelopmental and genetic disorders. The CDIs have been widely used in clinical populations as a screening tool (Bolton et al., 2012; Filipe et al., 2025), a tool for monitoring changes in expressive language skills over time (Charman et al., 2003; Foster-Cohen et al., 2023; Maassen et al., 2022), or as an outcome measure (Luyster et al., 2007; McDaniel et al., 2018; Sparaci et al., 2018). As summarized in the Results, the evidence across the included studies reveals several comparable patterns: (i) delayed onset of expressive vocabulary across all conditions, (ii) marked heterogeneity in individual growth trajectories (Figs. 6–8), and (iii) limited availability of multi-timepoint datasets, with most studies collecting only 1–2 measurements. The descriptive trajectories should be interpreted with caution, as age clusters combined studies using different CDI forms in different languages; therefore, differences between clusters may reflect both developmental change and variation of the study designs.

However, comparing these age trends across languages and forms remains challenging due to variation in CDI length and structure across forms (Words&Gestures, Words&Sentences, CDI-III, short forms) and cross-linguistic adaptations. These methodological differences limit our ability to conduct precise cross-condition or cross-linguistic comparisons. One possible solution could be the conversion of the CDI scores to proportions to eliminate differences in scale (Belteki et al., 2025). This approach would require the authors to report the descriptive data in a more detailed way or share the raw scores in public repositories. Unfortunately, many studies report only mean scores, without interquartile ranges, percentiles, or consistent documentation of which CDI version was used (see Supplementary Tables 1–4). Few papers offer subgroup analyses (e.g., different outcomes within elevated likelihood for adverse outcome samples), further limiting interpretability. As a result, we are constrained in our ability to synthesize or compare findings across studies, which makes it difficult to draw robust conclusions about condition-specific language development or perform cross-condition meta-analytical comparisons.

Nonetheless, the present review is informative for three reasons. First, by organizing evidence around within-condition developmental change, we can identify consistent patterns of delay, divergence, convergence, spurts, and plateaus that are robust to absolute scaling differences. Second, by systematically documenting sources of heterogeneity such as version length, scoring rules (e. g., inclusion of signed items), sampling frames, and timing, we can explain discrepant findings and provide interpretive guardrails for clinicians and researchers. Third, we outline a concrete harmonization pathway for future longitudinal work: (i) proportion-based scoring when item totals and descriptive statistics are reported; (ii) use of core overlapping item sets across forms/languages to anchor comparisons; (iii) standardized reporting of administration/analysis choices (age windows, version, adaptations, intelligibility considerations); and (iv) open data/data dictionaries to enable re-analysis and cross-cohort synthesis. We have predominantly focused on a descriptive approach, and in the following sections, we examine how CDIs have been applied to specific neurodevelopmental and genetic conditions.

### 4.1. Autism

Most studies that applied the CDIs in the context of neurodevelopmental conditions focused on autism. We were able to include 50 publications in our review (see Supplementary Table 1). However, data on expressive vocabulary could only be extracted from 25 publications (Fig. 5). The CDIs were used in the context of autism research across a wide range of ages (from 12 months to 6 years), starting at the end of the first year of life as deficits in language skills are apparent from the early phase in language acquisition in autistic individuals (Tager-Flusberg, 2016). The specific nature and extent of language skills in children with autism are variable and there is significant heterogeneity of language development trajectories in infants at elevated likelihood of autism (EL-infants). For example, in an Italian group of EL-infants studied at 12, 18, and 24 months, researchers identified four patterns of expressive vocabulary development: 18.2 % had above-average skills, 38.7 % developed language typically, 11.7 % had late-onset language development, and 31.4 % consistently showed a stable language delay (Riva et al., 2021). A delay in expressive vocabulary in children with autism was observed in comparison to the trajectories of low-likelihood children (Charman et al., 2003; Luyster et al., 2007) as well as EL-children who were not diagnosed with autism (Mitchell et al., 2006, see also a review in Belteki et al. 2022). Therefore, monitoring developmental trajectories of expressive language skills during the second year of life may be crucial for early identification of autism. This is particularly important in light of findings suggesting that a substantial proportion of autistic children experience a period of developmental regression (e.g., loss of previously acquired words or social communication behaviors; Clarke, 2019; Jones & Campbell, 2010; Pickles et al., 2022). Therefore, a multi-timepoint assessment of expressive vocabulary could be valuable to better understand developmental regression in autism (Boterberg et al., 2019). Longitudinal CDI data can help differentiate between children who show a slow but steadily increasing vocabulary, those who exhibit prolonged plateaus, and those who demonstrate stagnation or loss of previously acquired words.

In addition, emerging evidence highlights the role of camouflaging and gender-related differences in autism (Bölte et al., 2023). Because CDI scores rely on cumulative word production rather than momentary performance, repeated CDI assessments may be sensitive to subtle divergence patterns, such as slower growth rates, delayed spurts, or increasing discrepancies between expressive vocabulary and other communicative domains. These patterns may be especially relevant for girls or children with milder phenotypes, in whom expressive language abilities can mask broader social-communicative difficulties. While CDIs are not designed to measure camouflaging directly, their longitudinal use can contribute indirect markers of such effects when interpreted alongside clinical and behavioral data.

### 4.2. Down syndrome

While autism has been the primary focus of CDI use in neurodevelopmental conditions, several genetic syndromes have also been studied, with Down syndrome (DS) and Williams syndrome (WS) receiving the most attention. There are 28 publications on CDI used with participants with Down syndrome included in our review (see Supplementary Table 2). However, data on expressive vocabulary could only be extracted from 17 publications (Fig. 5). In children with DS (caused by the presence of a third copy of chromosome 21), expressive vocabulary development follows a markedly delayed but eventually verbal developmental trajectory. Initial delays in speech-language development have been widely documented (e.g., Berglund et al., 2001; Joyce & Dimitriou, 2017; Vanvuchelen et al., 2011; Thomas et al., 2020), prompting some researchers to extend the chronological age range of CDI assessments beyond the typical scope (Deckers et al., 2016). Given these early pervasive delays, several studies have expanded the CDI to include both spoken and signed vocabulary in a single score, to better reflect the importance of gesture use in DS relative to spoken language (Deckers et al., 2016). Children with DS are often taught signs drawn from sign languages or augmentative and alternative communication systems (Caselli et al., 1998; Deckers et al., 2016; Ellis et al., 2008; Foster-Cohen et al., 2023; Mervis & Robinson, 2000). The CDI studies have consistently shown that expressive vocabulary in DS lags well behind that of typically developing (TD) peers. For example, vocabulary sizes at ages 3 and 4 in DS were found to be comparable to those of TD children at just 16–20 months (Berglund et al., 2001). In addition, children with DS tend to produce more telegraphic and incomplete sentences than their TD peers (Volterra et al., 2003). Taken together, these findings underscore the importance of developmental timing in interpreting CDI data for children with DS. The tool can effectively track progress in early expressive vocabulary, but adaptations, such as extending age norms or including signed items, may be required to more accurately capture expressive vocabulary development in this group (Deckers et al., 2016). While many individuals with DS become increasingly verbal later in life, early assessments must account for the uneven developmental profile and potential underestimation of vocabulary due to intelligibility issues (Galeote et al., 2016). Thus, caution is warranted when comparing CDI results in DS to those from TD children, particularly when relying solely on parent-reported spoken word production.

### 4.3. Williams syndrome

Another genetic syndrome in which the CDIs have been applied is Williams syndrome (WS, caused by a deletion of chromosome 7q11.23). Only 8 publications related to this syndrome are present in our review (Supplementary Table 3 and Fig. 5). Toddlers with Williams syndrome (WS) also show different developmental trajectories of communicative development compared with TD children and children with other developmental conditions (e.g., Becerra et al., 2019; Vicari et al., 2002; Volterra et al., 2003). Many children with WS start using words to refer to objects several months before they begin pointing to share interest, and they also make unique types of grammatical errors not typically seen in TD children (Becerra et al., 2019; Volterra et al., 2003). Cross-syndrome comparisons between WS and DS show mixed results. While Harris et al. found that children with DS had larger expressive vocabulary sizes than children with WS at 2 years, Mervis & Robinson reported the opposite (Harris et al., 1997; Mervis & Robinson, 2000). They attributed the discrepancy to methodological limitations in the earlier study, including age mismatches between groups, narrow outcome measures, and insufficient attention to syndrome-specific developmental profiles. Overall, more multi-timepoint studies are needed to fully understand how expressive vocabulary unfolds in this population.

### 4.4. 5p deletion syndrome

The CDIs have also been used to assess language skills in case reports of children with 5p deletion syndrome (caused by a terminal deletion of the short arm of chromosome 5), also known as *Cri du chat* syndrome. However, for this syndrome, we were able to include only 2 publications in the review (Fig. 5). Findings suggest that although vocabulary development in these children is delayed, it tends to follow a trajectory similar to that of typically developing peers (Kristoffersen & Simonsen, 2025; Kristoffersen, 2020). However, even though the condition is often identified early due to atypical, high-pitched “cat-like” vocalizations in infancy (Lejeune et al., 1963), early speech and language development has not been systematically studied. As a result, little is known about how communication skills unfold over time in this population.

### 4.5. Angelman syndrome

Angelman syndrome (AS) is caused by loss of function of the ubiquitin–protein ligase E3A (*UBE3A*) gene, e.g., through maternal deletions of 15q11–13, paternal uniparental disomy, imprinting defects or single point mutations. Individuals with AS typically exhibit profound impairments in expressive and receptive language, often producing few or no functional spoken words, with reported vocabularies rarely exceeding 20 words (Jolleff & Ryan, 1993; Micheletti et al., 2016; Quinn & Rowland, 2017). Given this severe limitation in spoken language, the applicability of CDIs for assessing expressive vocabulary growth in AS is inherently restricted, which is reflected in the small number of available studies (3 identified in the present review; Fig. 5). For example, Micheletti et al. (2016) reported a floor effect for spoken word production across the entire sample, alongside markedly reduced receptive scores. Despite these limitations, CDIs may still provide informative insights when focusing on non-verbal communicative behaviors, particularly gesture use and communicative intent. Indeed, Jolleff et al. (2006) used CDI-based measures to explore phenotype differences associated with genetic mechanisms of AS, reporting higher communicative intent in individuals with non-deletion variants. These findings suggest that, while CDIs are poorly suited for tracking spoken vocabulary development in AS, gesture and communication-related items may offer a more sensitive window into early communicative profiles. From a comparative perspective, CDI data in AS contribute to cross-condition contrasts, illustrating the lower bounds of expressive language development relative to other neurodevelopmental and genetic conditions.

### 4.6. DDX3X syndrome

DDX3X syndrome (caused by a spontaneous mutation within the *DDX3X* gene) is a genetic syndrome that accounts for 1–3 % of cases of unexplained developmental delay and/or intellectual disability in females (Snijders Blok et al., 2015) and is also significantly associated with autism (Ruzzo et al., 2019; Takata et al., 2018; Yuen et al., 2017). In terms of speech-language skills, a prospective study (see Supplementary Table 4) showed that 80 % of children with DDX3X syndrome had atypical expressive vocabulary and 73 % had atypical receptive language. Parents reported in the CDIs that the average number of words understood by their child was 278 and words produced was 165 (note the wide range of age of participants: from 3 to 16 years), indicating higher receptive than expressive skills (Tang et al., 2021). In those with more advanced speech-language skills, the average age of the first word was 31 months, and the average age of the first multiword utterance was 48 months. The lack of speech or significant speech delays were also reported in other cohorts and case studies using various measures (e.g., Stefaniak et al., 2022; Levy et al., 2023; Parra et al., 2024, for a comprehensive review, see Stefaniak et al., 2023).

### 4.7. NRXN1 deletions

Another gene that is associated with intellectual disability and autism (as well as other neurodevelopmental and neuropsychiatric phenotypes) is *NRXN1* (Schaaf et al., 2012). Only 1 study (Supplementary Table 4; Fig. 5) described speech-language phenotypes (using the CDIs and other measures) in 21 children with exonic deletions of *NRXN1* and showed that 57 % of children demonstrated some degree of receptive and/or expressive language delay, but language abilities were highly variable ranging from non-verbal or severely delayed to above average (Brignell et al., 2018). No dominant speech and language patterns were consistently observed in the group of children with *NRXN1* deletions.

### 4.8. Other genetic syndromes: Rett syndrome, fragile X syndrome, tuberous sclerosis complex

In other genetic conditions, the CDIs have been applied in a variety of clinical contexts (Supplementary Table 4). For example, in a case study of a girl with Rett syndrome (linked to mutations in the *MECP2* gene), the CDIs were used to monitor receptive language development before and after treatment between the ages of 5 and 8 years (Pizzamiglio et al., 2008). In Fragile X syndrome (FXS; caused by trinucleotide repeat expansions in the 5’-untranslated region of the *FMR1* gene), Jackson and Roberts (1999) found that parents rated their children’s receptive—but not expressive—vocabulary higher than professionals, suggesting differences in perception or reporting styles. Using other parent-report measures, Hinton et al. (2013) similarly reported a marked delay in the production of first words, taking into account the presence or absence of comorbid autism symptoms in individuals with FXS. In addition, retrospective video analyses have been used to objectively identify and validate restricted communicative forms and functions in this population (Marschik et al., 2014). The CDI has also been used in studies of Tuberous Sclerosis Complex (caused by loss-of-function mutations in the *TSC1* or *TSC2* genes; Henske et al., 2016) to examine the relationship between expressive and receptive vocabulary, gesture use, and epilepsy-related clinical features. In this context, longer epilepsy duration and the use of multiple antiepileptic drugs were associated with a greater risk of language development delay (Foryś-Basiejko et al., 2022).

### 4.9. Cerebral palsy

The CDIs have also been used in studies on disorders that primarily affect motor functions (Supplementary Table 4). Foster-Cohen et al. (2023) showed a large variation in spoken/signed vocabulary size in children with cerebral palsy (CP, caused by damage that occurs to the developing brain) using a modified version of the CDIs with added sign option for each item. Based on CDI results, Viswanath et al. (2023) reported that 35.7 % of patients aged 2–18 years had no understanding of any communication and 46.4 % did not use any communicative gestures. In non-verbal children with CP, Molinaro et al. (2020) used the CDIs to longitudinally measure receptive vocabulary growth, showing that receptive vocabulary scores are increasing at a reduced pace for most children with CP (compared to TD) and that the size of children’s vocabulary at 48 months is a strong indicator of how quickly they continued to learn new words.

### 4.10. Spinal muscular atrophy type 1

Early language acquisition was also measured with the CDIs in children with spinal muscular atrophy type 1 (type 1 SMA), associated with homozygous deletions of the *SMN1* gene. Children diagnosed with type 1 SMA demonstrate severe oral motor weakness progressing to complete anarthria. Although faced with severe motor impairments, intelligence and cognition are usually unaffected in type 1 SMA (e.g., Kolb & Kissel, 2015). In a longitudinal cohort study with Italian participants (Buchignani et al., 2024) showed that at 8 months of age, comprehension skills were preserved in 81 % of the type 1 SMA but the expressive scores were < 5th percentile in more than 80 % of children and gesture abilities were < 5th percentile in 55 % of children with type 1 SMA. At follow-up (at 36 months), despite an increase in expressive vocabulary, the scores remained below the fifth percentile in 43 % type 1 SMA.

### 4.11. Sex chromosome trisomies

Sex chromosome trisomies are genetic conditions in which an individual possesses three sex chromosomes instead of the typical two. The three recognized forms include females with a 47,XXX karyotype, referred to as triple X syndrome (TX), and males with either a 47,XXY karyotype, known as Klinefelter syndrome (KS), or a 47,XYY karyotype, referred to as 47,XYY syndrome. Children with sex chromosome trisomies were assessed with the CDIs by Zampini et al. (2018a) at the age of 24 months and 60 % of them had expressive vocabulary size below the 5th percentile, suggesting a significant likelihood for language impairment. Their number of spontaneously produced words was lower than in TD children at the same age. With regard to KS specifically, Zampini et al. (2018b) reported that in an Italian cohort all 18-month-old children with KS had expressive vocabulary sizes lower than the 50th percentile of the Italian normative data, and 4 out of 13 children were below the 10th percentile.

### 4.12. Use of CDI in a cross-sectional vs. longitudinal study designs

Beyond documenting language phenotypes across conditions, several methodological considerations arise in how the CDIs are implemented and interpreted in clinical research. Monitoring expressive language development requires longitudinal studies with multiple measurement time points. Single-measurement approaches do not tell us much in terms of condition-specific speech-language development, particularly given the large variability in scores of children with neurodevelopmental disorders – especially considering the tendency of this variability to increase with age (the so-called fan effect, where individual differences become more pronounced over time; Fenson et al., 1994; Marschik et al., 2007; Kalinowski et al., 2025; Rescorla et al., 2000). Furthermore, the approach to correlate the scores from single measurement points in infancy or toddlerhood with later diagnosis outcomes does not allow for capturing the nonlinear trajectories such as language regression in autism or the initial delay followed by partial improvement described for Williams syndrome. Such patterns highlight the need for growth models that can accommodate both spurts and plateaus. In the last years, novel analytical approaches are emerging to better capture vocabulary growth curves from longitudinal and accelerated longitudinal designs (see Day et al., 2025 for a summary “growth score” modeling that accounts for ceiling and floor effects using Gompertz curves) that could aid more fine-grained analyses of speech-language acquisition.

### 4.13. Validity and reliability in the context of neurodevelopmental conditions

Beyond study design, the accuracy and consistency of the CDIs in clinical populations is another key area of concern. The validity and reliability of the CDIs across languages and versions remain uncertain and the strategies for assessing these parameters vary significantly between language versions (Jarůšková et al., 2023). Already 25 years ago, Feldman et al. (2000) criticized the CDIs as having too much variability, too little stability, and insufficient ability to predict early language delay. The large variability can be (at least partially) explained by the overall large variability in the rates of expressive language development in infancy and toddlerhood (e. g., Fenson et al., 2000). The analyses of concurrent validity between the CDIs and Mullen Scales of Early Learning (Mullen, 1995) in the context of autism yield mixed results, from moderate agreement in 14-month-olds (Belteki et al., 2025) to very high agreement in 24-month-olds (Nordahl-Hansen et al., 2014).

Another issue related to the parental reports are parental tendencies – observed and documented in relation to parents of typically-developing children – to under- or over-estimate their children’s accuracy in both comprehension and production tasks have been documented with differential reporting biases in different subgroups of the population (e.g., Feldman et al., 2000). Parental estimations seem to exceed children’s actual performance by approximately 10 % (in comprehension) to 20 % (in production) (Frank et al., 2021; Łuniewska et al., 2024). Furthermore, parents also deviate in their reporting of their child’s knowledge from test to retest (Mayor & Mani, 2019). In addition, while the parental checklist may exhibit high reliability and the overall scores derived from the checklists may correlate with the overall scores of direct measures of vocabulary (e.g., Sachse and Von Suchodoletz, 2008), the validity can still be low for individual test items. It can be particularly tricky for larger vocabulary sizes – the more words a child knows, the lower the agreement between parent report and direct measures (Łuniewska et al., 2024). This has direct implications in autism, where toddlers with restricted interests may develop highly specialized vocabularies that caregivers struggle to fully recall or that are not covered in the lexical item lists, leading to underestimation. Similarly, some studies indicate differences in the words that were most reported in CDIs by caregivers and the patterns of children’s word usage derived from samples of spontaneous language production during free play (Gatt et al., 2023). Furthermore, parents of children with neurodevelopmental conditions may not have the same reporting styles as parents of TD children (Galeote et al., 2016; Miller et al., 1995). For example, Miller et al. (1995) observed that parents of children with developmental delays tend to be either more conservative or more uncertain in their estimates of language skills, possibly due to limited expectations or lower confidence in their child’s abilities. Galeote et al. (2016) similarly noted that in the context of Down syndrome, parents may underreport spoken vocabulary if the child frequently relies on gestures or signs, or if speech intelligibility is low – highlighting the role of communication modality in shaping parental perceptions. This variability in parental interpretation, influenced by syndrome-specific characteristics or previous clinical feedback, can introduce bias or inconsistencies in cross-group comparisons. It also emphasizes the need for condition-sensitive adaptations of parent-report measures and, where possible, integration with clinician-administered assessments or observational tools. Moreover, in communities where it is common to have multiple caregiving systems including sibling caregiving, parents may under- or overestimate their child’s vocabulary size given that childcare is frequently provided by another caregiver (Vogt et al., 2015). Interestingly, Nordahl-Hansen et al. (2013) mention good inter-rater reliability between parents and preschool teachers’ language ratings in children with autism, which suggests that reports from different caregivers can be aligned.

Caregiver reports tend to be most accurate for children with language delays and smaller vocabularies, likely because these concerns lead parents to monitor development more closely (Bennetts et al., 2016). However, parents who perceive their child as having a more difficult temperament may underestimate their language abilities compared to direct assessments (Bennetts et al., 2016). This is particularly relevant for neurodevelopmental conditions like autism, where children often show distinct temperament traits—such as increased negative affect, lower extraversion, and reduced effortful control (Kerekes et al., 2013; Mallise et al., 2020). These traits may further complicate accurate parent-reported estimates of vocabulary size.

### 4.14. Screening potential of the CDIs for early language difficulties in neurodevelopmental disorders and genetic conditions

The potential of the CDIs to serve as early screening tools for language delays and deviations in neurodevelopmental conditions has received growing attention. Screening refers to the early and quick identification of new cases at risk for disease or disorder to refer the individuals for further evaluation. Given their parent-report format, ease of administration, and coverage of early vocabulary and communication milestones, CDIs have long been considered promising candidates for screening efforts (Jago et al., 2023; Kim et al., 2014; Kim et al., 2016; Westerlund et al., 2006). A review by Eriksson (2023) showed that there is insufficient evidence that the CDIs are a valid tool for screening for language difficulties, as no study could convincingly show that the actual diagnostic accuracy was sufficient for clinical use. However, Filipe et al. (2025) reported promising sensitivity for a purpose-built screening version of the CDI in European Portuguese, which effectively identified children performing below the 10th percentile. Furthermore, Holzinger et al. (2022) demonstrated good predictability of language development (one year after screening) and high feasibility of a strongly reduced word-list based on the CDI (in Austrian German) combined with other variables (e.g. word combinations, word comprehension, parental concerns). Still, these findings remain preliminary and require replication in other populations and languages. There have also been initial efforts to design condition-specific adaptations of the CDI. For example, Galeote et al. (2016) developed CDI-Down (European Spanish) aimed at children with DS by combining the WG and WS forms into a single inventory and incorporating the assessment of symbolic and referential gestures. These modifications are intended to address the fact that the chronological age of children with DS often significantly exceeds their developmental language age, and that gesture and sign use is a characteristic strength in this population.

Importantly, the age at which a neurodevelopmental condition is typically diagnosed may also influence parental reporting styles. Children with DS are commonly identified at birth or prenatally, which allows parents to monitor development closely from the start. In contrast, autism is often diagnosed much later, and early signs may not be easily recognized by caregivers. These differences in parental awareness and expectations may impact how language abilities are reported in the CDI. Thus, while emerging adaptations show promise, the current consensus remains that CDIs, at least in their standard form, are not yet sufficiently validated for broad, cross-condition diagnostic screening.

Despite the growing use of CDIs in neurodevelopmental research and applied contexts, most studies are still limited by small sample sizes, heterogeneous administration procedures, and a lack of cross-linguistic standardization and normative data. Another notable limitation of the current literature – and of our review – is the limited consideration of bi-/multilingual development in neurodevelopmental conditions (but see examples in Bird et al., 2005; Hambly & Fombonne, 2012; Petersen et al., 2012). Going forward, CDIs work in clinical populations should systematically document language exposure, and develop bilingual norms/adaptations where feasible. This is critical for equitable assessment in linguistically diverse settings.

To fully understand how language develops in atypically developing infants and toddlers, it is essential to acknowledge the complexity of developmental processes. This includes integrating data from multiple domains and timepoints, and capturing both within- and between-syndrome variability (Karmiloff-Smith et al., 2012; Karmiloff-Smith, 1998; Karmiloff-Smith, 2009; D’Souza et al., 2017; D’Souza & D’Souza, 2022, 2024; Marschik et al., 2017; Sansavini et al., 2021). Future applications of the CDIs in cross-cultural neurodevelopmental research will require not only linguistically and culturally sensitive adaptations but also reflection on condition-specific strengths and challenges. Digital formats of the CDI (e.g., Web-CDI and mobile apps) may help facilitate this process by enabling scalable, low-burden, and longitudinal data collection—particularly in clinical and cross-cultural settings.

## 5. Conclusions

Taken together, the patterns summarized in the Results and elaborated in the Discussion reveal both shared and condition-specific developmental signatures: all groups show delayed onset relative to TD norms, yet the shape of growth (e.g., gradual vs. plateaued trajectories, gesture–speech trade-offs) varies substantially across conditions. These empirical trends motivate our call for harmonized, multi-timepoint longitudinal designs to deepen the understanding of these patterns. The Communicative Development Inventories (CDIs) are a valuable tool for assessing early expressive and receptive language in children with neurodevelopmental and genetic conditions. While they do not replace direct clinical assessments, CDIs can serve as reliable, low-burden instruments for establishing developmental baselines, screening and tracking progress over time, and informing early intervention efforts, particularly in under-resourced or culturally diverse settings. However, despite growing use, the application of CDIs to neurodevelopmental populations remains fragmented, and condition-specific pathways of early language development have not been systematically studied.

The CDIs utility is maximized when used within longitudinal frameworks. Single-timepoint assessments often fail to capture the high variability of non-linear trajectories frequently observed in neurodevelopmental conditions. This is further complicated by differences in the timing of diagnosis: some populations (e.g., Down syndrome) are recognized before the onset of lexical development, enabling prospective data collection, whereas others (e.g., autism) are typically diagnosed after this window, limiting early measurements. Combined with the marked clinical heterogeneity across conditions, these differences make it challenging to compare developmental profiles from first words through later speech-language outcomes.

The condition-specific expressive vocabulary age trends visualized in this review for the three conditions most studied in terms of early language development as assessed by CDIs (autism, DS and WS) offer an important lens into the divergence and convergence of developmental trajectories, and they highlight the value of CDI data in characterizing these paths. Yet these comparisons are challenging because of variability in language adaptation processes, scoring protocols, and condition-specific factors that can affect parent-report accuracy and comparability across studies.

Future work should prioritize improving the validity and cultural and disorder-specific adaptability of CDI tools, harmonizing scoring protocols and reporting schemes across languages, and supporting open data sharing to enable cross-linguistic and cross-condition comparisons. With continued methodological refinement and broader accessibility, CDIs can play an increasingly important role in shaping equitable, early language assessment practices worldwide.

## Supplementary Material

1

## Data Availability

No data was used for the research described in the article.

## Appendix A. Supporting information

Supplementary data associated with this article can be found in the online version at doi:10.1016/j.ridd.2026.105256.
