# Supplementary material for: Communicative Development Inventories (CDIs) in etiologically diverse developmental conditions: A systematic review
Source: Res Dev Disabil. Author manuscript; Available in PMC 2026 May 27. (PMC13215705; doi:10.1016/j.ridd.2026.105256)
Supplement: 1 [file NIHMS2177846-supplement-1.docx]

**Supplementary Materials**

**Supplementary Table 1**

| **Autism** | | | | | | | | | | | | |
| --- | --- | --- | --- | --- | --- | --- | --- | --- | --- | --- | --- | --- |
| **Paper** | | **Participants** | | | | **Scores** | | | | | **CDI** | |
| Authors | Year | Population | Sample size | Mean age [in months] | Age range [in months] | Mean score | Min score | Max score | Percentile | SD | CDI version | Language |
| Abdelaziz et al. | 2018 | autism diagnosis | 29 | 33.00 | 18 to 42 | 52.86 | n.r. | n.r. | n.r. | 70.90 | W&G (8 to 16 months), only CDI nouns | English |
| Abdi et al. | 2023 | autism diagnosis - T0 | 10 | 47.90 | n.r. | 27.80 | n.r. | n.r. | n.r. | 13.06 | CDI 1 - Infant form | Persian |
|  |  | autism diagnosis - T1 | 10 | 49.90 | n.r. | 93.40 | n.r. | n.r. | n.r. | 27.24 | CDI 1 - Infant form | Persian |
|  |  | autism diagnosis - T2 | 10 | 51.90 | n.r. | 144.90 | n.r. | n.r. | n.r. | 34.58 | CDI 1 - Infant form | Persian |
| Auza-Benavides et al. | 2024 | autism risk | 41 | 24.39 | 18 to 30 | n.r. | n.r. | n.r. | 50%: 47; 25%: 3; 75%: 160.5 | n.r. | CDI II W&S | Spanish |
| Charman et al. | 2003 | autism diagnosis | 134 | 38.00 | 18 to 88 | 38.70 | n.r. | n.r. | n.r. | 68.70 | CDI Infant Form W&G | English |
| Ellawadi and Weismer | 2014 | autism diagnosis | 78 | 30.51 | 23 to 37 | 39.35 | n.r. | n.r. | n.r. | 67.50 | W&G | English |
| Gengoux | 2019 | autism diagnosis - W&G - T0 | 22 | 44.60 | 24 to 72 | 165.00 | n.r. | n.r. | n.r. | 141.90 | W&G, words produced out of 396 | English |
|  |  | autism diagnosis - W&G - T1 | 22 | 47.60 | 24 to 72 | 204.90 | n.r. | n.r. | n.r. | 154.70 | W&G, words produced out of 396 | English |
|  |  | autism diagnosis - W&S - T0 | 22 | 44.6 | 24 to 72 | 238.6 | n.r. | n.r. | n.r. | 210.60 | W&S, words produced out of 680 | English |
|  |  | autism diagnosis - W&S - T1 | 22 | 47.60 | 24 to 72 | 310.30 | n.r. | n.r. | n.r. | 237.30 | W&S, words produced out of 680 | English |
| Hambly and Fombonne | 2014 | autism diagnosis, NON-B | 10 | 60.30 | 36 to 84 | 366.00 | n.r. | n.r. | n.r. | 176.00 | W&S | English, Quebec French, Spanish, Hebrew, Chinese, Romanian |
|  |  | autism diagnosis, LOW-B | 11 | 59.60 |  | 440.00 | n.r. | n.r. | n.r. | 160.00 | W&S | English, Quebec French, Spanish, Hebrew, Chinese, Romanian |
|  |  | Autism diagnosis, HIGH-B | 12 | 60.40 |  | 569.00 | n.r. | n.r. | n.r. | 103.00 | W&S | English, Quebec French, Spanish, Hebrew, Chinese, Romanian |
| Holzinger et al. | 2019 | autism diagnosis, ESDM - T0 | 7 | 42.30 | 34 to 54 | 81.70 | n.r. | n.r. | n.r. | 75.30 | A-CDI 2 | Austrian German |
|  |  | autism diagnosis, ESDM - T1 | 7 | 54.30 |  | 324.70 | n.r. | n.r. | n.r. | 201.90 | A-CDI 2 | Austrian German |
|  |  | autism diagnosis, usual intervention - T0 | 6 | 44.50 |  | 33.00 | n.r. | n.r. | n.r. | 58.80 | A-CDI 2 | Austrian German |
|  |  | autism diagnosis, usual intervention - T1 | 6 | 56.50 |  | 193.80 | n.r. | n.r. | n.r. | 229.50 | A-CDI 2 | Austrian German |
| Hudry et al. | 2010 | Likely or confirmed autism diagnosis | 152 | 44.83 | 24 to 59 | 100.40 | 0 | 391 | n.r. | 121.20 | MCDI Infant form, 396 words; scores for sample size 147 | English |
| Kjellmer et al. | 2012 | autism diagnosis - total | 129 | 45.00 | 24 to 63 | n.r. | n.r. | n.r. | n.r. | n.r. | W&G + W&S (partly) | Swedish |
|  |  | autism unspecified | 11 | n.r. | n.r. | 300.60 | n.r. | n.r. | n.r. | 96.60 | W&G + W&S (partly) | Swedish |
|  |  | PDD-NOS | 32 | n.r. | n.r. | 182.30 | n.r. | n.r. | n.r. | 127.70 | W&G + W&S (partly) | Swedish |
|  |  | AD | 78 | n.r. | n.r. | 93.30 | n.r. | n.r. | n.r. | 113.60 | W&G + W&S (partly) | Swedish |
|  |  | Asperger syndrome | 8 | n.r. | n.r. | 356.90 | n.r. | n.r. | n.r. | 21.20 | W&G + W&S (partly) | Swedish |
| Kover and Ellis Weismer | 2014 | autism diagnosis, no. of coded words max. 140 | 57 | 30.35 | 21 to 37 | 90.82 | 10 | 299 | n.r. | 79.77 | W&S | English |
|  |  | autism diagnosis, no. of coded words 20 - 140 | 31 | 30.70 | 21 to 37 | 144.10 | 43 | 299 | n.r. | 72.95 | W&S | English |
| Lorang et al. | 2023 | autism diagnosis | 21 | 42.81 | 26 to 71 | 116.19 | 0 | 322 | n.r. | 118.20 | W&G (total=396 words) + custom vocabulary checklist from MCDI-WG | English |
| Luyster et al. | 2007 | autism diagnosis total | 93 | 34.67 | 8 to 72 | n.r. | n.r. | n.r. | n.r. | n.r. | W&G and earlier composite version of W&G + W&S, only items appearing in both used for analysis | English |
|  |  | autism | 66 | n.r. | n.r. | 24.01 | n.r. | n.r. | n.r. | 69.72 | W&G and earlier composite version of W&G + W&S, only items appearing in both used for analysis | English |
|  |  | PDD-NOS | 27 | n.r. | n.r. | 102.52 | n.r. | n.r. | n.r. | 120.77 | W&G and earlier composite version of W&G + W&S, only items appearing in both used for analysis | English |
| McDaniel et al. | 2018 | autism diagnosis - T0 | 65 | 43.46 | 32 to 56 | 17.00 | 0 | 117 | n.r. | 25.00 | W&G | English |
|  |  | autism diagnosis - T1 | 65 | 51.36 | 40 to 64 | 88.00 | 0 | 296 | n.r. | 101.00 | W&G | English |
| McDuffie and Yoder | 2010 | Autism diagnosis (29 autistic, 3 PDD-NOS) - T0 | 32 | 40.65 | 27 to 60 | 52.96 | 0 | 232 | n.r. | 65.75 | W&G (8 to 16 months) | English |
|  |  | autism diagnosis (29 autistic, 3 PDD-NOS) - T1 | 32 | 46.63 | 32 to 67 | 105.19 | 0 | 343 | n.r. | 107.26 | W&G (8 to 16 months) | English |
| Mitchell et al. | 2006 | autism sibling - T0 | 15 | 13.00 | 11 to 15 | 2.20 | 0 | 12 | n.r. | 3.19 | CDI - Infant form, W&G, 396 words | English |
|  |  | autism sibling - T1 | 15 | 18.65 | 18 to 20 | 21.12 | 0 | 130 | n.r. | 34.10 | CDI - Infant form, W&G, 396 words | English |
| Nazari et al. | 2025 | autism diagnosis - total | 1539 | 28.60 | n.r. | n.r. | n.r. | n.r. | n.r. | n.r. | WG & WS | English |
|  |  | autism diagnosis - male - WG | 1200 | 14.90 | 9,6 to 18.5 | 64.00 | n.r. | n.r. | n.r. | n.r. | 176 as sample size | English |
|  |  | autism diagnosis - male - WS | 1200 | 24.80 | 15,6 to 30,5 | 64.00 | n.r. | n.r. | n.r. | n.r. | 482 as sample size | English |
|  |  | autism diagnosis - female - WG | 339 | 15.20 | 12,1 to 17,7 | 62.60 | n.r. | n.r. | n.r. | n.r. | 55 as sample size | English |
|  |  | autism diagnosis - female - WS | 339 | 24.40 | 18,0 to 30,5 | 62.60 | n.r. | n.r. | n.r. | n.r. | 141 as sample size | English |
| Nordahl-Hansen et al. | 2013 | autism diagnosis - parent rating | 55 | 48.70 | 29 to 60 | 118.40 | 0 | 364 | n.r. | 120.60 | W&G, 396 words | Norwegian |
|  |  | autism diagnosis - preschool teachers' rating | 55 | 48.70 | 29 to 60 | 106.80 | 0 | 342 | n.r. | 115.60 | W&G, 396 words | Norwegian |
| Sandbank et al. | 2017 | ASD diagnosis | 34 | 45.40 | 25,53 to 62,29 | 44.76 | 0 | 254 | n.r. | 74.25 | W&G | English |
| Schafer et al. | 2013 | autism diagnosis | 26 | 82.20 | 27,5 to 150 | 231.00 | 12 | 691 | n.r. | 237.00 | W&S - modified | English |
| Sheppard et al. | 2017 | autism risk - total - T0 | 31 | 30.20 | 18 to38 | 36.00 | 2.5 | 100 | n.r. | 33.20 | short form of W&G (8 to 18 months) and W&S (16 to 30 months) --> estimates for long form included in paper | English |
|  |  | autism risk - total - T1 | 31 | 33.20 |  | 42.90 | 9.5 | 100 | n.r. | 31.40 | short form of W&G (8 to 18 months) and W&S (16 to 30 months) --> estimates for long form included in paper | English |
|  |  | autism risk - Treatment - T0 | 14 | 30.80 |  | 40.70 | n.r. | n.r. | n.r. | 37.70 | short form of W&G (8 to 18 months) and W&S (16 to 30 months) | English |
|  |  | autism risk - Treatment - T1 | 12 | 33.90 |  | 45.80 | n.r. | n.r. | n.r. | 33.40 | short form of W&G (8 to 18 months) and W&S (16 to 30 months) | English |
|  |  | autism risk - Placebo - T0 | 13 | 29.60 |  | 31.00 | n.r. | n.r. | n.r. | 28.20 | short form of W&G (8 to 18 months) and W&S (16 to 30 months) | English |
|  |  | autism risk - Placebo - T1 | 12 | 32.40 |  | 39.70 | n.r. | n.r. | n.r. | 30.40 | short form of W&G (8 to 18 months) and W&S (16 to 30 months) | English |
| Sparaci et al. | 2018 | EL infants --> received autism diagnosis later | 11 | n.r. | n.r. | 12.10 | n.r. | n.r. | n.r. | 14.15 | CDI-III (30 to 37 months) | English |
| Su et al. | 2018 | autism diagnosis - total | 160 | 49.73 | 12 to 72 | n.r. | n.r. | n.r. | n.r. | n.r. | PCDI W&S | Mandarin |
|  |  | Low verbal | 95 | 46.07 | 17 to 83 | 44.38 | 0 | 229 | n.r. | 64.32 | PCDI W&S | Mandarin |
|  |  | Middle verbal | 39 | 52.90 | 28 to 76 | 388.23 | 265 | 498 | n.r. | 68.64 | PCDI W&S | Mandarin |
|  |  | High verbal | 26 | 58.35 | 32 to 81 | 662.12 | 518 | 790 | n.r. | 77.92 | PCDI W&S | Mandarin |
| Tran et al. | 2020 | autism concern | 14 | n.r. | n.r. | 9.10 | 0 | 37 | n.r. | 11.80 | W&G | English |
| Whitehouse et al. | 2019 | autism signs - usual intervention - T0 | 53 | 12.38 | 9 to 14 | 1.11 | n.r. | n.r. | 50%: 0.00; 25%: 0.00; 75%: 1.25 | 2.05 | W&G, 396 words | English |
|  |  | autism signs - usual intervention - T1 | 53 | 18.38 | 15 to 20 | 16.63 | n.r. | n.r. | 50%: 8.00; 25%: 2.00; 75%: 20.00 | 27.59 | W&G, 396 words | English |
|  |  | autism signs - iBASIS-VIP - T0 | 50 | 12.40 | 9 to 14 | 1.24 | n.r. | n.r. | 50%: 0.00; 25%: 0.00; 75%: 1.00 | 2.25 | W&G, 396 words | English |
|  |  | autism signs - iBASIS-VIP - T1 | 50 | 18.40 | 15 to 20 | 27.22 | n.r. | n.r. | 50%: 12.00; 25%: 1.2; 75%: 28.25 | 43.22 | W&G, 396 words | English |

Supplementary Table S1. Summary of publications using the CDI (expressive vocabulary) on study samples with autism, or at elevated risk for a later autism diagnosis, focussing on publication year, study population, sample size, age (mean, range), mean expressive vocabulary (mean, minimum and maximum scores, percentile and standard deviation), CDI version and language.

**Supplementary Table 2**

| **Down syndrome** | | | | | | | | | | | | |
| --- | --- | --- | --- | --- | --- | --- | --- | --- | --- | --- | --- | --- |
| **Paper** | | **Participants** | | | | **Scores** | | | | | **CDI** | |
| Authors | Year | Population | Sample size | Mean age [in months] | Age range [in months] | Mean score | Min score | Max score | Percentile | SD | CDI version | Language |
| Bello A, Onofrio D, Caselli MC | 2014 | DS | 14 | 54.00 | 34-73 | 230.60 | 58 | 407 |  | 99.80 | W&G | Italian |
| Bird EK, Cleave P, Trudeau N, Thordardottir E, Sutton A, Thorpe A. | 2005 | DS monoling | 14 | 73.90 | 31-101 | 262.10 | n.r. | n.r. | n.r. | 72.40 | n.r. + sign | English, French |
|  |  | DS biling | 8 | 85.50 | 55-137 | 265.00 | n.r. | n.r. | n.r. | 121.70 | n.r. + sign | English, French |
| Caselli MC, Vicari S, Longobardi E, Lami L, Pizzoli C, Stella G. | 1998 | DS | 40 | 28.30 | 10-49 | 26.50 | 0 | 302 | n.r. | 55.50 | W&G | Italian |
| Deckers SR, Van Zaalen Y, Mens EJ, Van Balkom H, Verhoeven L. | 2016 | DS | 25 | 55.00 | 29-87 | 155.50 | 0 | 697 | n.r. | 203.30 | modified W&S (added extra column for signed words) | Dutch |
|  |  | DS | 25 | 55.00 | 29-87 | 91.90 | 0 | 301 | n.r. | 85.90 | modified W&S (added extra column for signed words) | Dutch |
|  |  | DS | 25 | 73.00 | 47-105 | 304.90 | 0 | 623 | n.r. | 196.00 | modified W&S (added extra column for signed words) | Dutch |
|  |  | DS | 25 | 73.00 | 47-105 | 84.10 | 0 | 351 | n.r. | 118.00 | modified W&S (added extra column for signed words) | Dutch |
| Dulin MS, Loveall SJ, Mattie LJ. | 2023 | DS | 13 | 17.92 | 11-29 | 6.08 | 0 | 25 | n.r. | 7.54 | W&G | English |
|  |  | DS | 10 | 24.30 | 16-48 | 15.10 | 0 | 66 | n.r. | 21.02 | W&G | English |
| Foster-Cohen S, Macrae T, Newbury J. | 2023 |  | 35 | 49.15 | 30-66 | 172.63 | n.r. | n.r. | n.r. | 126.21 | W&S + sign | English |
| Foster-Cohen S, Newbury J, Macrae T, van Bysterveldt A. | 2022 | DS | 35 | 49.68 | 36-66 | 179.07 | 6 | 595 | n.r. | 122.68 | W&S + sign | English |
| Galeote M, Checa E, Sánchez-Palacios C, Sebastián E, Soto P. | 2016 | DS | 29 | 52.12 | 26,4-72,19 | 275.59 | 18 | 640 | n.r. | 181.92 | CDI-Down | Spanish |
| Harris NGS, Bellugi U, Bates E, Jones W, Rossen M | 1997 | DS | 39 | 39.00 | 12-76 | 150.00 | n.r. | n.r. | n.r. | 172.00 | W&G + W&S | English |
| Joyce A, Dimitriou D. | 2017 | DS | 22 | 36.57 | 24,38-56,48 | 18.55 | n.r. | n.r. | n.r. | 1.16 | W&G | English |
| Mervis CB, Robinson BF. | 2000 | DS | 28 | 30.00 | 24-35.4 | 66.35 | 0 | 324 | n.r. | 79.24 | W&S | English |
| Schafer G, Williams TI, Smith PT | 2013 | DS | 28 | 51.40 | 23.7-133 | 249.00 | 20 | 699 | n.r. | 210.00 | W&S - modified | English |
| Singh SJ, Gan B, Chu SY | 2021 | DS | 25 | 25.60 | 12-36 | 138.52 | 6 | 315 | n.r. | 111.04 | W&G | English, Bahasa Melayu, Mandarin Chinese |
| Zampini L, D'Odorico L. | 2009 | DS | 20 | 36.00 | 35,93-38,03 | n.r. | 0 | 243 | n.r. |  | n.r. | Italian |
|  |  | DS | 20 | 42.00 | 41,93-44,03 | n.r. | 0 | 499 | n.r. |  | n.r. | Italian |
| Zampini L, Salvi A, D'Odorico L. | 2015 | DS | 18 | 24.00 | 24-26 | 8.83 | 0 | 21 | n.r. | 5.31 | W&G | Italian |
|  |  | DS | 18 | 30.00 | 30-32 | 22.44 | 2 | 85 | n.r. | 18.67 | W&G | Italian |
| Vicari, S., Caselli, M. C., Gagliardi, C., Tonucci, F., & Volterra, V. | 2002 | DS | 12 | 67.20 | n.r. | 457.00 | n.r. | n.r. | n.r. | 125.40 | W&S | Italian |
| Volterra, V., Caselli, M. C., Capirci, O., Tonucci, F., & Vicari, S. | 2003 | DS | 6 | 58.00 | n.r. | 428.00 | 298 | 521 | n.r. | 80.80 | W&S (670 words) | Italian |

Supplementary Table S2. Summary of publications using the CDI (expressive vocabulary) on study samples with Down syndrome focussing on publication year, study population, sample size, age (mean, range), mean expressive vocabulary (mean, minimum and maximum scores, percentile and standard deviation), CDI version and language.

**Supplementary Table 3**

| **Williams syndrome** | | | | | | | | | | | | |
| --- | --- | --- | --- | --- | --- | --- | --- | --- | --- | --- | --- | --- |
| **Paper** | | **Participants** | | | | **Scores** | | | | | **CDI** | |
| Authors | Year | Population | Sample size | Mean age [in months] | Age range [in months] | Mean score | Min score | Max score | Percentile | SD | CDI version | Language |
| Becerra and Mervis | 2019 | WS diagnosis - T0 | 47 | 24.44 | 23,95 to 24,94 | 43.21 | 0 | 176 | 50%: 22.00; 25%: 4.00; 75%: 41.00 | 41.89 | W&S, 680 words | English |
|  |  | WS diagnosis - T1 | 47 | 48.50 | 47,97 to 49,00 | 388.72 | 4 | 679 | 50%: 423.00; 25%: 196.00; 75%: 569.00 | 220.93 | W&S, 680 words | English |
| Greiner de Magalhaes et al. | 2020 | WS diagnosis | 96 | ~27 | 24 to 36 | 99.50 | 84 | 130 | 50%: 98; 25%: 90.5; 75%: 108 | 11.04 | W&S, 680 words | English |
| Mervis and Robinson | 2000 | WS diagnosis | 24 | ~30 | ~24 to 35 | 132.50 | 3 | 391 | n.r. | 122.29 | W&S | English |
|  |  | WS diagnosis - younger sample | 13 | ~25 | ~24 to 27 | 55.08 | 5 | 120 | n.r. | 40.80 | W&S | English |
| Laing et al. | 2002 | WS diagnosis - experiment 1 | 13 | 31.00 | 17 to 55 | 56.00 | n.r. | n.r. | n.r. | 83.30 | W&G | English |
|  |  | WS diagnosis - experiment 2 | 11 | 29.60 | n.r. | 55.60 | n.r. | n.r. | n.r. | 89.50 | W&G | English |
| Axelsson et al. | 2013 | WS diagnosis | 14 | 31.67 | 18.50 to 48.20 | 120.71 | 7 | 345 | n.r. | 137.76 | W&G (8 to 30 months) | English |
| Harris et al. | 1997 | WS diagnosis - total | 69 | 41.00 | 12 to 76 | 217.00 | n.r. | n.r. | n.r. | 222.00 | W&G (18 to 16 months), 396 words + W&S (16 to 30 months), 689 words | English |
|  |  | WS diagnosis - W&G | 34 | 34.00 | n.r. | 77.00 | n.r. | n.r. | n.r. | 101.00 | W&G (18 to 16 months), 396 words + W&S (16 to 30 months), 689 words | English |
|  |  | WS diagnosis - W&S | 35 | 47.00 | n.r. | 366.00 | n.r. | n.r. | n.r. | 208.00 | W&G (18 to 16 months), 396 words + W&S (16 to 30 months), 689 words | English |
| Volterra et al. | 2003 | WS diagnosis | 6 | 53.00 | n.r. | 430.00 | n.r. | n.r. | n.r. | 137.70 | PVB words and phrases, 670 words, 18 to 30 months | Italian |
| Vicari et al. | 2002 | WS diagnosis | 12 | 58.20 | n.r. | 452.00 | n.r. | n.r. | n.r. | 157.30 | PVB words and phrases | Italian |

Supplementary Table S3. Summary of publications using the CDI (expressive vocabulary) on study samples with Williams syndrome focussing on publication year, study population, sample size, age (mean, range), mean expressive vocabulary (mean, minimum and maximum scores, percentile and standard deviation), CDI version and language.

**Supplementary Table 4**

| **Other conditions** | | | | | | | | | | | | |
| --- | --- | --- | --- | --- | --- | --- | --- | --- | --- | --- | --- | --- |
| **Paper** |  | **Participants** |  |  |  | **Scores** |  |  |  |  | **CDI** |  |
| Authors | Year | Population | Sample size | Mean age [in months] | Age range [in months] | Mean score | Min score | Max score | Percentile | SD | CDI version | Language |
| Foster-Cohen S, Macrae T, Newbury J. | 2023 | CP | 8 | 45.18 | 30-66 | 345.24 | n.r. | n.r. | n.r. | 280.40 | W&S, words produced out of 675 | English |
| Ko EJ, Hong MJ, Choi EJ, Yuk JS, Yum MS, Sung IY. | 2021 | CP | 13 | 75.40 | 37-120 | 194.40 | n.r. | n.r. | n.r. | 255.80 | n.r. | Korean |
| Jackson, S. C., & Roberts, J. E. | 1999 | Fragile X syndrome | 16 | 44.80 | 20-74 | 217.50 | n.r. | n.r. | n.r. | 249.24 | W&G (8-16m, 396 words), W&S (16-30m, 680 words) | English |
| Tang, L., Levy, T., Guillory, S., Halpern, D., Zweifach, J., Giserman-Kiss, I., Foss-Feig, J. H., Frank, Y., Lozano, R., Belani, P., Layton, C., Lerman, B., Frowner, E., Breen, M. S., De Rubeis, S., Kostic, A., Kolevzon, A., Buxbaum, J. D., Siper, P. M., & Grice, D. E. | 2021 | DDX3X | 15 | 89.00 | 36-192 | 165.00 | n.r. | n.r. | n.r. | 186.10 | W&G (396 words) | English |
| Zampini, L., Burla, T., Silibello, G., Dall’Ara, F., Rigamonti, C., Lalatta, F., & Vizziello, P. | 2018 | Klinefelter syndrome | 13 | 17.92 | 17-19 | 9.83 | 0 | 32 | <50 | 9.04 | W&S | Italian |

Supplementary Table S4. Summary of publications using the CDI (expressive vocabulary) on study samples with other conditions (Cerebral palsy, Fragile X syndrome, DDX3X, Klinefelter syndrome) focusing on publication year, study population, sample size, age (mean, range), mean expressive vocabulary (mean, minimum and maximum scores, percentile and standard deviation), CDI version and language. Studies displayed assessed expressive vocabulary in relation to the different conditions across varying sample sizes. For more detailed information on case studies, see Laudańska et al. (in press)
